# Supplementary material for: Strain-induced nonlinear spin Hall effect in topological Dirac semimetal
Source: Sci Rep. 2018 Oct 15;8:15236. doi: 10.1038/s41598-018-33655-w (PMC6189066; doi:10.1038/s41598-018-33655-w)
Supplement: Supplementary file 1 — Supplemental Information [file 41598_2018_33655_MOESM1_ESM.pdf]

Supplemental Information for "Strain-induced nonlinear spin Hall effect in topological Dirac semimetal"

Yasufumi Araki[1,2]

[1] Institute for Materials Research, Tohoku University, Sendai 980-8577, Japan
[2] Frontier Research Institute for Interdisciplinary Sciences, Tohoku University, Sendai 980-8578, Japan

In this Supplemental Information, we show the details of our calculation, especially the solution process of the Boltzmann equation in response to the electric field. We here employ the perturbative expansion by $\boldsymbol{B}_5$ and neglect the terms beyond $O(B_5^1)$ in all the equations shown below.

Appendix A: Fourier decomposition of the Boltzmann equation

In order to estimate the response to the alternating electric field $\boldsymbol{E}(t) = \boldsymbol{E}_0(e^{i\omega t} + e^{-i\omega t})$, we here decompose the single-particle equations of motions

$$\dot{\boldsymbol{r}} = \boldsymbol{\nabla}_{\boldsymbol{k}} \tilde{\epsilon}_{s\eta}(\boldsymbol{k}) - \dot{\boldsymbol{k}} \times \boldsymbol{\Omega}_{s\eta}(\boldsymbol{k}) \tag{A1}$$

$$\dot{\boldsymbol{k}} = -e\boldsymbol{E}_\eta - e\dot{\boldsymbol{r}} \times \boldsymbol{B}_\eta \tag{A2}$$

and the Boltzmann equation

$$\left[\dot{\boldsymbol{r}} \cdot \boldsymbol{\nabla}_{\boldsymbol{r}} + \dot{\boldsymbol{k}} \cdot \boldsymbol{\nabla}_{\boldsymbol{k}} + \partial_t\right] f_{s\eta}(\boldsymbol{r}, \boldsymbol{k}, t) = (df_{s\eta}/dt)_{\mathrm{coll}} \tag{A3}$$

into the Fourier components with the frequencies $0,\ \pm\omega,\ \pm 2\omega, \cdots$.

By solving Eqs. (A1) and (A2) for $\dot{\boldsymbol{r}}$ and $\dot{\boldsymbol{k}}$, we obtain

$$D_{s\eta}\dot{\boldsymbol{r}} = \tilde{\boldsymbol{v}}_{s\eta} + e\boldsymbol{E}_\eta \times \boldsymbol{\Omega}_{s\eta} + e(\tilde{\boldsymbol{v}}_{s\eta} \cdot \boldsymbol{\Omega}_{s\eta})\boldsymbol{B}_\eta \tag{A4}$$

$$D_{s\eta}\dot{\boldsymbol{k}} = -e\boldsymbol{E}_\eta - e\tilde{\boldsymbol{v}}_{s\eta} \times \boldsymbol{B}_\eta - e^2(\boldsymbol{E}_\eta \cdot \boldsymbol{B}_\eta)\boldsymbol{\Omega}_{s\eta}, \tag{A5}$$

where $\tilde{\boldsymbol{v}}_{s\eta}(\boldsymbol{k}) \equiv \boldsymbol{\nabla}_{\boldsymbol{k}}\tilde{\epsilon}_{s\eta}(\boldsymbol{k})$ denotes the group velocity modified by the orbital magnetization. The momentum-space Berry curvature $\boldsymbol{\Omega}_{s\eta}(\boldsymbol{k})$ and the orbital magnetic moment $\boldsymbol{m}_{s\eta}(\boldsymbol{k})$ for the eigenstate $u_{s\eta}(\boldsymbol{k})$ are defined by

$$\boldsymbol{\Omega}_{s\eta}(\boldsymbol{k}) = -\mathrm{Im}\,\langle \boldsymbol{\nabla}_{\boldsymbol{k}} u_{s\eta}(\boldsymbol{k})| \times |\boldsymbol{\nabla}_{\boldsymbol{k}} u_{s\eta}(\boldsymbol{k})\rangle \tag{A6}$$

$$\boldsymbol{m}_{s\eta}(\boldsymbol{k}) = -\frac{e}{2}\mathrm{Im}\,\langle \boldsymbol{\nabla}_{\boldsymbol{k}} u_{s\eta}| \times [H_{s\eta}(\boldsymbol{k}) - \epsilon(\boldsymbol{k})]\,|\boldsymbol{\nabla}_{\boldsymbol{k}} u_{s\eta}\rangle\,, \tag{A7}$$

respectively. As the electromagnetic fields here read

$$\boldsymbol{E}_\eta(t) = \boldsymbol{E}_0(e^{i\omega t} + e^{-i\omega t}), \quad \boldsymbol{B}_\eta = \eta\boldsymbol{B}_5, \tag{A8}$$

the right-hand sides of Eqs. (A4) and (A5) can be decomposed into Fourier components as

$$D_{s\eta}\dot{\boldsymbol{r}} = \sum_{\nu=0,\pm1} e^{i\nu\omega t}\boldsymbol{R}_{s\eta}^{(\nu\omega)} \tag{A9}$$

$$D_{s\eta}\dot{\boldsymbol{k}} = \sum_{\nu=0,\pm1} e^{i\nu\omega t}\boldsymbol{K}_{s\eta}^{(\nu\omega)}, \tag{A10}$$

with

$$\boldsymbol{R}_{s\eta}^{(0)} = \tilde{\boldsymbol{v}}_{s\eta} + e(\tilde{\boldsymbol{v}}_{s\eta} \cdot \boldsymbol{\Omega}_{s\eta})\boldsymbol{B}_\eta \tag{A11}$$

$$\boldsymbol{R}_{s\eta}^{(\pm\omega)} = e\boldsymbol{E}_0 \times \boldsymbol{\Omega}_{s\eta} \tag{A12}$$

$$\boldsymbol{K}_{s\eta}^{(0)} = -e\tilde{\boldsymbol{v}}_{s\eta} \times \boldsymbol{B}_\eta \tag{A13}$$

$$\boldsymbol{K}_{s\eta}^{(\pm\omega)} = -e\boldsymbol{E}_0 - e^2(\boldsymbol{E}_0 \cdot \boldsymbol{B}_\eta)\boldsymbol{\Omega}_{s\eta}. \tag{A14}$$

Here the superscript in $(\cdot)$ on each physical quantity denotes its oscillation frequency.

Similarly, we also apply the Fourier decomposition to the time-dependent distribution function $f_{s\eta}(\boldsymbol{k}, t)$, which is given by

$$f_{s\eta}(\boldsymbol{k}, t) = \sum_{n\in\mathbb{Z}} e^{in\omega t} f_{s\eta}^{(n\omega)}(\boldsymbol{k}). \tag{A15}$$

By using these Fourier components, the Boltzmann equation

$$\left[\dot{\boldsymbol{k}} \cdot \boldsymbol{\nabla}_{\boldsymbol{k}} + \partial_t\right] f_{s\eta}(\boldsymbol{k}, t) = -\tau^{-1}\left[f_{s\eta}(\boldsymbol{k}, t) - f_{s\eta}^{\mathrm{eq}}(\boldsymbol{k})\right], \tag{A16}$$

which is reduced from Eq. (A3) by the homogeneous and relaxation-time approximations, can be decomposed as

$$\left[\left(\boldsymbol{K}_{s\eta}^{(0)} \cdot \boldsymbol{\nabla}_{\boldsymbol{k}}\right) + D_{s\eta}\tau_n^{-1}\right] f_{s\eta}^{(n\omega)} - D_{s\eta}\tau^{-1} f_{s\eta}^{\mathrm{eq}}\delta_{n0} + \left(\boldsymbol{K}_{s\eta}^{(+\omega)} \cdot \boldsymbol{\nabla}_{\boldsymbol{k}}\right) f_{s\eta}^{(n^-\omega)} + \left(\boldsymbol{K}_{s\eta}^{(-\omega)} \cdot \boldsymbol{\nabla}_{\boldsymbol{k}}\right) f_{s\eta}^{(n^+\omega)} = 0, \tag{A17}$$

with $\tau_n^{-1} \equiv \tau^{-1} + in\omega$ and $n^\pm \equiv n \pm 1$, for each frequency $n\omega$. Each equation correlates three neighboring Fourier components of the distribution function; by solving these equations iteratively, we can estimate the distribution function at any frequency. Since $\boldsymbol{K}_{s\eta}^{(0)}$ is independent of $\boldsymbol{E}_0$ and $\boldsymbol{K}_{s\eta}^{(\pm\omega)}$ is linear in $\boldsymbol{E}_0$, we can easily see from Eq. (A17) that $f_{s\eta}^{(n\omega)}$ is of $O(E_0^{|n|})$ for small E_0.

Once we obtain the solution for those equations, we can evaluate the Fourier components of the current for each spin and valley as

$$\boldsymbol{j}_{s\eta}^{(n\omega)} = -e\int \frac{d^3\boldsymbol{k}}{(2\pi)^3}\left[\boldsymbol{R}_{s\eta}^{(0)} f_{s\eta}^{(n\omega)} + \sum_{\pm} \boldsymbol{R}_{s\eta}^{(\pm\omega)} f_{s\eta}^{(n^\mp\omega)}\right], \tag{A18}$$

which is based on the relation

$$\boldsymbol{j}_{s\eta}(t) = -e\int \frac{d^3\boldsymbol{k}}{(2\pi)^3} D_{s\eta}(\boldsymbol{k})\dot{\boldsymbol{r}} f_{s\eta}(\boldsymbol{k}, t). \tag{A19}$$

In particular, the stationary (DC) current can be given as

$$\boldsymbol{j}_{s\eta}^{(0)} = -e \int \frac{d^3\boldsymbol{k}}{(2\pi)^3} \left[\boldsymbol{R}_{s\eta}^{(0)} f_{s\eta}^{(0)} + \sum_{\pm} \boldsymbol{R}_{s\eta}^{(\pm\omega)} f_{s\eta}^{(\mp\omega)} \right], \tag{A20}$$

which we shall use later to estimate the rectified current.

Appendix B: Useful formulae in polar coordinate

Before solving the Boltzmann equation iteratively, we first list up some formula useful in solving the Boltzmann equation under the Dirac Hamiltonian. Here we take the terms up to the second order in $\boldsymbol{E}_0$ and the linear order in $\boldsymbol{B}_5$.

We mainly use the polar coordinate (k, θ, ϕ) in the momentum space, defined by

$$\boldsymbol{k} = k\left(\hat{\boldsymbol{e}}_x \sin\theta\cos\phi + \hat{\boldsymbol{e}}_y \sin\theta\sin\phi + \hat{\boldsymbol{e}}_z\cos\theta\right). \tag{B1}$$

The basis vectors in this coordinate are defined by

$$\hat{\boldsymbol{k}} = \hat{\boldsymbol{e}}_x \sin\theta\cos\phi + \hat{\boldsymbol{e}}_y \sin\theta\sin\phi + \hat{\boldsymbol{e}}_z\cos\theta \tag{B2}$$

$$\hat{\boldsymbol{e}}_\theta = \hat{\boldsymbol{e}}_x \cos\theta\cos\phi + \hat{\boldsymbol{e}}_y \cos\theta\sin\phi - \hat{\boldsymbol{e}}_z\sin\theta \tag{B3}$$

$$\hat{\boldsymbol{e}}_\phi = -\hat{\boldsymbol{e}}_x \sin\phi + \hat{\boldsymbol{e}}_y\cos\phi, \tag{B4}$$

and the gradient operator is given by

$$\boldsymbol{\nabla}_{\boldsymbol{k}} = \hat{\boldsymbol{k}}\partial_k + \hat{\boldsymbol{e}}_\theta \frac{1}{k}\partial_\theta + \hat{\boldsymbol{e}}_\phi \frac{1}{k\sin\theta}\partial_\phi. \tag{B5}$$

We should note the following formulae for gradient,

$$\boldsymbol{\nabla}_{\boldsymbol{k}} k = \hat{\boldsymbol{k}} \tag{B6}$$

$$\boldsymbol{\nabla}_{\boldsymbol{k}} a_\parallel = \frac{1}{k}\boldsymbol{a}_\perp \tag{B7}$$

with $\boldsymbol{a}$ independent of $\boldsymbol{k}$. Here $a_\parallel$ and $\boldsymbol{a}_\perp$ are decomposition of $\boldsymbol{a}$ into the components parallel and perpendicular to $\boldsymbol{k}$, respectively, which are explicitly defined by

$$a_\parallel \equiv \boldsymbol{a}\cdot\hat{\boldsymbol{k}}, \quad \boldsymbol{a}_\perp \equiv \boldsymbol{a} - a_\parallel\hat{\boldsymbol{k}}. \tag{B8}$$

We also list up several formulae for the angular integral $\int d\Omega = \int d\theta \ \sin\theta \ d\phi$,

$$\int d\Omega \ \hat{\boldsymbol{k}} = 0 \tag{B9}$$

$$\int d\Omega \ a_\parallel \hat{\boldsymbol{k}} = \frac{4\pi}{3}\boldsymbol{a} \tag{B10}$$

$$\int d\Omega \ a_\parallel b_\parallel \hat{\boldsymbol{k}} = 0 \tag{B11}$$

$$\int d\Omega \ a_\parallel^2 b_\parallel \hat{\boldsymbol{k}} = \frac{4\pi}{15}\left[3(\boldsymbol{a}\cdot\boldsymbol{b})\boldsymbol{a} - (\boldsymbol{b}\times\boldsymbol{a})\times\boldsymbol{a}\right]. \tag{B12}$$

The radial integral of the delta functions can be evaluated as

$$\int_0^\infty dk \ k^n \delta(k - k_{\mathrm{F}}) = k_{\mathrm{F}}^n \tag{B13}$$

$$\int_0^\infty dk \ k^n \delta'(k - k_{\mathrm{F}}) = -\int_0^\infty dk \ nk^{n-1}\delta(k - k_{\mathrm{F}}) \tag{B14}$$

$$= -nk_{\mathrm{F}}^{n-1}. \tag{B15}$$

Appendix C: Explicit forms for Dirac/Weyl dispersion

For a Weyl electron in the electron band, the Berry curvature and the orbital magnetic moment, defined by Eqs. (A6) and (A7), are given by

$$\boldsymbol{\Omega}_{s\eta}(\boldsymbol{k}) = s\eta\frac{1}{2k^2}\hat{\boldsymbol{k}}, \quad \boldsymbol{m}_{s\eta}(\boldsymbol{k}) = s\eta\frac{ev_{\mathrm{F}}}{2k}\hat{\boldsymbol{k}}. \tag{C1}$$

Thus the phase space volume $D_{s\eta}$ and the modified single-particle energy $\tilde{\epsilon}_{s\eta}$ reduce to η-independent forms,

$$D_{s\eta}(\boldsymbol{k}) = 1 + e\boldsymbol{B}_\eta\cdot\boldsymbol{\Omega}_{s\eta}(\boldsymbol{k}) = 1 + s\frac{eB_{5\parallel}}{2k^2} \tag{C2}$$

$$\tilde{\epsilon}_{s\eta}(\boldsymbol{k}) = \epsilon(\boldsymbol{k}) - \boldsymbol{m}_{s\eta}(\boldsymbol{k})\cdot\boldsymbol{B}_\eta \tag{C3}$$

$$= v_{\mathrm{F}}k - s\frac{ev_{\mathrm{F}}}{2k}B_{5\parallel}. \tag{C4}$$

The modified group velocity $\tilde{\boldsymbol{v}}_{s\eta}$ also becomes η-independent, given as

$$\tilde{\boldsymbol{v}}_{s\eta}(\boldsymbol{k}) = \boldsymbol{\nabla}_{\boldsymbol{k}}\tilde{\epsilon}_{s\eta}(\boldsymbol{k}) \tag{C5}$$

$$= v_{\mathrm{F}}\hat{\boldsymbol{k}} + s\frac{ev_{\mathrm{F}}}{2k^2}B_{5\parallel}\hat{\boldsymbol{k}} - s\frac{ev_{\mathrm{F}}}{2k^2}\boldsymbol{B}_{5\perp} \tag{C6}$$

$$= \left(1 + s\frac{eB_{5\parallel}}{k^2}\right)v_{\mathrm{F}}\hat{\boldsymbol{k}} - s\frac{ev_{\mathrm{F}}}{2k^2}\boldsymbol{B}_5. \tag{C7}$$

The equilibrium distribution under the axial magnetic field $\boldsymbol{B}_5$ is given as

$$f_{s\eta}^{\mathrm{eq}}(\boldsymbol{k}) = \theta\left(\mu - \tilde{\epsilon}_{s\eta}(\boldsymbol{k})\right) \tag{C8}$$

$$= \theta\left(k_{\mathrm{F}} - k + s\frac{e}{2k}B_{5\parallel}\right) \tag{C9}$$

$$\simeq \theta(k_{\mathrm{F}} - k) + s\frac{e}{2k}B_{5\parallel}\delta(k - k_{\mathrm{F}}), \tag{C10}$$

with the Fermi momentum $k_{\mathrm{F}} = \mu/v_{\mathrm{F}}$, which is not perfectly symmetric around the DP due to the orbital magnetization.

$\boldsymbol{R}_{s\eta}^{(\nu\omega)}$ and $\boldsymbol{K}_{s\eta}^{(\nu\omega)}$ defined by Eqs. (A12) and (A14) read

$$\boldsymbol{R}_{s\eta}^{(0)} = \left(1 + s\frac{eB_{5\parallel}}{k^2}\right)v_{\mathrm{F}}\hat{\boldsymbol{k}} \tag{C11}$$

$$\boldsymbol{R}_{s\eta}^{(\pm\omega)} = s\eta e\frac{1}{2k^2}\boldsymbol{E}_0\times\hat{\boldsymbol{k}} \tag{C12}$$

$$\boldsymbol{K}_{s\eta}^{(0)} = -\eta e v_{\mathrm{F}}\hat{\boldsymbol{k}}\times\boldsymbol{B}_5 \tag{C13}$$

$$\boldsymbol{K}_{s\eta}^{(\pm\omega)} = -e\boldsymbol{E}_0 - se^2\frac{\boldsymbol{E}_0\cdot\boldsymbol{B}_5}{2k^2}\hat{\boldsymbol{k}}, \tag{C14}$$

up to $O(B_5^1)$.

Appendix D: Solution of Boltzmann equation

1. Linear response

We first derive the linear response to the electric field, which is represented by $f_{s\eta}^{(\pm\omega)}$. It can be obtained by solving Eq. (A17) for $n=\pm 1$, which can be explicitly written as

$$\left[\left(\boldsymbol{K}_{s\eta}^{(0)}\cdot\boldsymbol{\nabla}_{\boldsymbol{k}}\right)+D_{s\eta}\tau_{\pm}^{-1}\right]f_{s\eta}^{(\pm\omega)}=-\left(\boldsymbol{K}_{s\eta}^{(\pm\omega)}\cdot\boldsymbol{\nabla}_{\boldsymbol{k}}\right)f_{s\eta}^{\rm eq}, \tag{D1}$$

up to the linear order in $\boldsymbol{E}_0$. Moving the first term on the left-hand side to the right-hand side, this equation can be rewritten as

$$f_{s\eta}^{(\pm\omega)}=F_{s\eta}^{(\pm\omega){\rm a}}+F_{s\eta}^{(\pm\omega){\rm b}} \tag{D2}$$

with

$$F_{s\eta}^{(\pm\omega){\rm a}}=-D_{s\eta}^{-1}\tau_{\pm}\left(\boldsymbol{K}_{s\eta}^{(\pm\omega)}\cdot\boldsymbol{\nabla}_{\boldsymbol{k}}\right)f_{s\eta}^{\rm eq} \tag{D3}$$

$$F_{s\eta}^{(\pm\omega){\rm b}}=-D_{s\eta}^{-1}\tau_{\pm}\left(\boldsymbol{K}_{s\eta}^{(0)}\cdot\boldsymbol{\nabla}_{\boldsymbol{k}}\right)f_{s\eta}^{(\pm\omega)}. \tag{D4}$$

which can be evaluated iteratively by substituting $f_{s\eta}^{(\pm\omega)}$ obtained on the left-hand side of Eq. (D2) to the right-hand side of Eq. (D4). Since $\boldsymbol{K}_{s\eta}^{(0)}$ is linear in $\boldsymbol{B}_5$, each iteration yields a term higher by B_5^1. We should make a single iteration to reach $f_{s\eta}^{(\pm\omega)}$ up to $O(B_5^1)$.

The first term $F_{s\eta}^{(\pm\omega){\rm a}}$ can be given by evaluating

$$\begin{aligned}F_{s\eta}^{(\pm\omega){\rm a}}\simeq{}&\tau_{\pm}\left(1-\frac{se}{2k^2}B_{5\parallel}\right)\\&\times\left[e\boldsymbol{E}_0+\frac{se^2}{2k^2}(\boldsymbol{E}_0\cdot\boldsymbol{B}_5)\hat{\boldsymbol{k}}\right]\cdot\boldsymbol{\nabla}_{\boldsymbol{k}}f_{\eta}^{\rm eq},\end{aligned} \tag{D5}$$

where we drop the terms higher than $O(B_5^1)$. The gradient of $f_{s\eta}^{\rm eq}$ reads

$$\begin{aligned}\boldsymbol{\nabla}_{\boldsymbol{k}}f_{\eta}^{\rm eq}&=\boldsymbol{\nabla}_{\boldsymbol{k}}\left[\theta(k_{\rm F}-k)+s\frac{e}{2k}B_{5\parallel}\delta(k-k_{\rm F})\right]\\&=\left[-\hat{\boldsymbol{k}}+\frac{se}{2k^2}\boldsymbol{B}_5-\frac{se}{k^2}B_{5\parallel}\hat{\boldsymbol{k}}+\frac{se}{2k}B_{5\parallel}\hat{\boldsymbol{k}}\partial_k\right]\delta_{\rm F},\end{aligned} \tag{D6}$$

where we employ shorthand notations

$$\delta_{\rm F}\equiv\delta(k-k_{\rm F}),\quad\delta'_{\rm F}\equiv\partial_k\delta(k-k_{\rm F}),\quad\cdots. \tag{D7}$$

By collecting the terms up to $O(B_5^1)$, we obtain

$$F_{s\eta}^{(\pm\omega){\rm a}}=-e\tau_{\pm}E_{0\parallel}\delta_{\rm F}-\frac{se^2\tau_n}{2k^2}B_{5\parallel}E_{0\parallel}(\delta_{\rm F}-k\delta'_{\rm F}) \tag{D8}$$

Since $\boldsymbol{K}_{\eta}^{(0)}$ is of $O(B_5^1)$, $f_{s\eta}^{(\pm\omega)}$ at $O(B_5^0)$ can be simply given as

$$f_{s\eta}^{(\pm\omega)}=-e\tau_{\pm}E_{0\parallel}\delta(k-k_{\rm F})+O(B_5^1) \tag{D9}$$

arising from $F_{s\eta}^{(\pm\omega){\rm a}}$. Therefore, $F_{s\eta}^{(\pm\omega){\rm b}}$ up to $O(B_5^1)$ can be obtained from Eq. (D4) as

$$\begin{aligned}F_{s\eta}^{(\pm\omega){\rm b}}&\simeq-D_{s\eta}^{-1}\tau_{\pm}\left(\boldsymbol{K}_{s\eta}^{(0)}\cdot\boldsymbol{\nabla}_{\boldsymbol{k}}\right)F_{s\eta}^{(\pm\omega){\rm a}}\big|_{\boldsymbol{B}_5=0} &&\text{(D10)}\\&\simeq-\eta e^2v_{\rm F}\tau_{\pm}^2\left(\hat{\boldsymbol{k}}\times\boldsymbol{B}_5\right)\cdot\boldsymbol{\nabla}_{\boldsymbol{k}}\left[E_{0\parallel}\delta_{\rm F}\right] &&\text{(D11)}\\&=-\eta e^2v_{\rm F}\tau_{\pm}^2\left(\hat{\boldsymbol{k}}\times\boldsymbol{B}_5\right)\cdot\left[\frac{1}{k}\boldsymbol{E}_{0\perp}\delta_{\rm F}+E_{0\parallel}\delta'_{\rm F}\hat{\boldsymbol{k}}\right] &&\text{(D12)}\\&=-\eta e^2v_{\rm F}\tau_{\pm}^2\left(\hat{\boldsymbol{k}}\times\boldsymbol{B}_5\right)\cdot\boldsymbol{E}_0\frac{1}{k}\delta_{\rm F} &&\text{(D13)}\\&=-\eta e^2v_{\rm F}\tau_{\pm}^2\frac{1}{k}\left(\boldsymbol{B}_5\times\boldsymbol{E}_0\right)_{\parallel}\delta_{\rm F}, &&\text{(D14)}\end{aligned}$$

where one should note that $\hat{\boldsymbol{k}}\times\boldsymbol{B}_5$ is perpendicular to $\hat{\boldsymbol{k}}$.

By collecting Eqs. (D8) and (D14), $f_{\eta}^{(\pm\omega)}(\boldsymbol{k})$ reads

$$\begin{aligned}f_{s\eta}^{(\pm\omega)}(\boldsymbol{k})={}&-e\tau_{\pm}E_{0\parallel}\delta_{\rm F}-\eta\frac{e^2v_{\rm F}\tau_{\pm}^2}{k}(\boldsymbol{B}_5\times\boldsymbol{E}_0)_{\parallel}\delta_{\rm F}\\&-s\frac{e^2\tau_{\pm}}{2k^2}E_{0\parallel}B_{5\parallel}(\delta_{\rm F}-k\delta'_{\rm F}),\end{aligned} \tag{D15}$$

up to $O(E_0^1,B_5^1)$. The first term in $[\cdots]$ represents the shift of the distribution from the drift by $\boldsymbol{E}(t)$, while the second term gives the shift from the regular Hall effect by the *axial* magnetic field $\boldsymbol{B}_5$ and the *normal* electric field $\boldsymbol{E}(t)$. The third term has a complicated structure: it gives a *quadrupolar* distribution in $\boldsymbol{k}$-space unless $\boldsymbol{E}_0$ and $\boldsymbol{B}_5$ are exactly parallel. It arises as the modification to the first (drift) term from the Berry curvature and the orbital magnetic moment. This term represents the effect of the chiral anomaly: it modifies the electron density, whose deviation from equilibrium is given by

$$\begin{aligned}\delta\rho_{s\eta}(t)={}&\int\frac{d^3\boldsymbol{k}}{(2\pi)^3}D_{s\eta}(\boldsymbol{k})\sum_{\pm}e^{\pm i\omega t}f_{s\eta}^{(\pm\omega)}(\boldsymbol{k}) &&\text{(D16)}\\&-\frac{se^2}{4\pi^2}\frac{2\tau}{1+\omega^2\tau^2}\boldsymbol{E}_0\cdot\boldsymbol{B}_5(\cos\omega t+\omega\tau\sin\omega t), &&\text{(D17)}\end{aligned}$$

where we have used

$$\int\frac{d^3\boldsymbol{k}}{(2\pi)^3}D_{s\eta}(\boldsymbol{k})f_{s\eta}^{(\pm\omega)}(\boldsymbol{k})=-s\frac{e^2\tau_{\pm}}{4\pi^2}\boldsymbol{E}_0\cdot\boldsymbol{B}_5. \tag{D18}$$

We can see that the induced electron density $\delta\rho_{s\eta}(t)$ for each valley/spin satisfies the continuity equation

$$\left[\partial_t+\frac{1}{\tau}\right]\delta\rho_{s\eta}(t)=-s\frac{e^2}{4\pi^2}\boldsymbol{B}_5\cdot\boldsymbol{E}(t), \tag{D19}$$

modified by the chiral anomaly, noting that $\boldsymbol{E}(t)=2\boldsymbol{E}_0\cos\omega t$. Although there is no spin flip term in the original Hamiltonian (except for the $\boldsymbol{k}^3$ terms), the anomaly violates spin conservation if $\boldsymbol{B}_5\cdot\boldsymbol{E}_0\neq 0$, leading to the spin polarization oscillating with the frequency ω.

2. Quadratic response

Next we solve Eq. (A17) for $n = 0$ to evaluate the stationary distribution $f_{s\eta}^{(0)}$ up to the second order in $\boldsymbol{E}_0$. The equation can be explicitly written as

$$\left[\left(\boldsymbol{K}_{s\eta}^{(0)} \cdot \boldsymbol{\nabla}_{\boldsymbol{k}}\right) + D_{s\eta}\tau^{-1}\right] \delta f_{s\eta}^{(0)} = -\sum_{\pm} \left(\boldsymbol{K}_{s\eta}^{(\mp\omega)} \cdot \boldsymbol{\nabla}_{\boldsymbol{k}}\right) f_{s\eta}^{(\pm\omega)}, \tag{D20}$$

where $\delta f_{s\eta}^{(0)} \equiv f_{s\eta}^{(0)} - f_{s\eta}^{\mathrm{eq}}$. Similarly to Eq. (D2), this equation can be rewritten as

$$\delta f_{s\eta}^{(0)} = F_{s\eta}^{(0)\mathrm{a}} + F_{s\eta}^{(0)\mathrm{b}} \tag{D21}$$

with

$$F_{s\eta}^{(0)\mathrm{a}} = -D_{s\eta}^{-1}\tau \sum_{\pm} \left(\boldsymbol{K}_{s\eta}^{(\mp\omega)} \cdot \boldsymbol{\nabla}_{\boldsymbol{k}}\right) f_{s\eta}^{(\pm\omega)} \tag{D22}$$

$$F_{s\eta}^{(0)\mathrm{b}} = -D_{s\eta}^{-1}\tau \left(\boldsymbol{K}_{s\eta}^{(0)} \cdot \boldsymbol{\nabla}_{\boldsymbol{k}}\right) \delta f_{s\eta}^{(0)}, \tag{D23}$$

which can again be evaluated iteratively.

Evaluation of $F_{s\eta}^{(0)\mathrm{a}}$ is lengthy but straightforward. The gradient of $f_{s\eta}^{(\pm\omega)}$ is given as

$$\begin{aligned}
\boldsymbol{\nabla}_{\boldsymbol{k}} f_{s\eta}^{(\pm\omega)} &= -e\tau_n \left(\frac{1}{k}\boldsymbol{E}_{0\perp}\delta_{\mathrm{F}} + \hat{\boldsymbol{k}}\delta'_{\mathrm{F}}\right) \\
&+ \frac{se^2\tau_n}{k^3} B_{5\parallel}E_{0\parallel}\hat{\boldsymbol{k}} \left(\delta_{\mathrm{F}} - k\delta'_{\mathrm{F}} + \frac{k^2}{2}\delta''_{\mathrm{F}}\right) \\
&- \frac{se^2\tau_n}{2k^3} \left(\boldsymbol{B}_{5\perp}E_{0\parallel} + B_{5\parallel}\boldsymbol{E}_{0\perp}\right)\left(\delta_{\mathrm{F}} - k\delta'_{\mathrm{F}}\right) \\
&- \frac{\eta e^2 v_{\mathrm{F}}\tau_n^2}{k^2} \left[(\boldsymbol{B}_5 \times \boldsymbol{E}_0)\delta_{\mathrm{F}} + (\boldsymbol{B}_5 \times \boldsymbol{E}_0)_{\parallel}(-2\delta_{\mathrm{F}} + k\delta'_{\mathrm{F}})\right].
\end{aligned} \tag{D24}$$

Substituting this form into Eq. (D22) and collecting the terms up to $O(B_5^1)$, we obtain

$$\begin{aligned}
F_{s\eta}^{(0)\mathrm{a}} = &-\frac{2e^2\tau\tau_*}{k} \left[(E_0^2 - E_{0\parallel}^2)\delta_{\mathrm{F}} + E_{0\parallel}^2 k\delta'_{\mathrm{F}}\right] \\
&+ \frac{se^3\tau\tau_*}{k^3} \Big[-(\boldsymbol{E}_0 \cdot \boldsymbol{B}_5)E_{0\parallel}\delta_{\mathrm{F}} + E_0^2 B_{0\parallel}k\delta'_{\mathrm{F}} \\
&\qquad + E_{0\parallel}^2 B_{5\parallel}(3\delta_{\mathrm{F}} - 3k\delta'_{\mathrm{F}} + 2k^2\delta''_{\mathrm{F}})\Big] \\
&+ \frac{2\eta e^3 v_{\mathrm{F}}\tau\tau_{**}^2}{k^2}(\boldsymbol{B}_5 \times \boldsymbol{E}_0)_{\parallel}E_{0\parallel}(2\delta_{\mathrm{F}} - k\delta'_{\mathrm{F}}),
\end{aligned} \tag{D25}$$

where τ_* and τ_{**} are defined as

$$\tau_* = \frac{1}{2}\left(\tau_+ + \tau_-\right) = \tau\frac{1}{1+\omega^2\tau^2} \tag{D26}$$

$$\tau_{**}^2 = \frac{1}{2}\left(\tau_+^2 + \tau_-^2\right) = \tau^2\frac{1-\omega^2\tau^2}{(1+\omega^2\tau^2)^2}. \tag{D27}$$

Since $\boldsymbol{K}_{\eta}^{(0)}$ is of $O(B_5^1)$, $\delta f_{s\eta}^{(0)}$ at $O(B_5^0)$ is given by the first line in Eq. (D25). Therefore, by substituting this part to Eq. (D23), we obtain

$$F_{s\eta}^{(0)\mathrm{b}} \simeq -D_{s\eta}^{-1}\tau \left(\boldsymbol{K}_{s\eta}^{(0)} \cdot \boldsymbol{\nabla}_{\boldsymbol{k}}\right) \delta F_{s\eta}^{(0)\mathrm{a}}\big|_{\boldsymbol{B}_5=0} \tag{D28}$$

$$\begin{aligned}
&\simeq -2\eta e^3 v_{\mathrm{F}}\tau^2\tau_*(\hat{\boldsymbol{k}} \times \boldsymbol{B}_5) \\
&\qquad \cdot \boldsymbol{\nabla}_{\boldsymbol{k}} \left[(E_0^2 - E_{0\parallel}^2)k^{-1}\delta_{\mathrm{F}} + E_{0\parallel}^2\delta'_{\mathrm{F}}\right] \\
&= -2\eta e^3 v_{\mathrm{F}}\tau^2\tau_*(\hat{\boldsymbol{k}} \times \boldsymbol{B}_5) \cdot E_{0\parallel}\boldsymbol{E}_0 \left[-\frac{2}{k^2}\delta_{\mathrm{F}} + \frac{2}{k}\delta'_{\mathrm{F}}\right]
\end{aligned} \tag{D29}$$

$$= \frac{4\eta e^3 v_{\mathrm{F}}\tau^2\tau_*}{k^2}(\boldsymbol{B}_5 \times \boldsymbol{E}_0)_{\parallel}E_{0\parallel}(\delta_{\mathrm{F}} - k\delta'_{\mathrm{F}}). \tag{D30}$$

Collecting Eqs. (D25) and (D30), we obtain the stationary response $\delta f_{s\eta}^{(0)}$.

Appendix E: Stationary current

Finally, we calculate the nonlinear stationary current from the distribution functions obtained above. From Eq. (A20), we split the stationary current $\boldsymbol{j}_{s\eta}^{(0)}$ into three parts,

$$\boldsymbol{j}_{s\eta}^{(0)} = \boldsymbol{j}_{s\eta}^{(0)\mathrm{eq}} + \boldsymbol{j}_{s\eta}^{(0)\mathrm{a}} + \boldsymbol{j}_{s\eta}^{(0)\mathrm{b}}, \tag{E1}$$

with

$$\boldsymbol{j}_{s\eta}^{(0)\mathrm{eq}} = -\frac{e}{(2\pi)^3}\int d^3\boldsymbol{k}\ \boldsymbol{R}_{s\eta}^{(0)}(\boldsymbol{k})f_{s\eta}^{\mathrm{eq}}(\boldsymbol{k}) \tag{E2}$$

$$\boldsymbol{j}_{s\eta}^{(0)\mathrm{a}} = -\frac{e}{(2\pi)^3}\int d^3\boldsymbol{k}\ \boldsymbol{R}_{s\eta}^{(0)}(\boldsymbol{k})\delta f_{s\eta}^{(0)}(\boldsymbol{k}) \tag{E3}$$

$$\boldsymbol{j}_{s\eta}^{(0)\mathrm{b}} = -\frac{e}{(2\pi)^3}\int d^3\boldsymbol{k}\ \sum_{\pm}\boldsymbol{R}_{s\eta}^{(\pm\omega)}(\boldsymbol{k})f_{s\eta}^{(\mp\omega)}(\boldsymbol{k}). \tag{E4}$$

Here $\boldsymbol{j}_{s\eta}^{(0)\mathrm{eq}}$ accounts for the equilibrium current that is present even in the absence of the electric field $\boldsymbol{E}(t)$, whereas the other two are nonequilibrium current driven by the electric field; $\boldsymbol{j}_{s\eta}^{(0)\mathrm{a}}$ comes from the stationary distribution $f_{s\eta}^{(0)}$ and the drift velocity $\boldsymbol{R}_{s\eta}^{(0)}$, while $\boldsymbol{j}_{s\eta}^{(0)\mathrm{b}}$ is from the oscillating distribution $f_{s\eta}^{(\mp\omega)}$ and the anomalous velocity $\boldsymbol{R}_{s\eta}^{(\pm\omega)}$.

The equilibrium current $\boldsymbol{j}_{s\eta}^{(0)\mathrm{eq}}$ can be straightforwardly evaluated as

$$\begin{aligned}
\boldsymbol{j}_{s\eta}^{(0)\mathrm{eq}} &= -\frac{ev_{\mathrm{F}}}{(2\pi)^3}\int d^3\boldsymbol{k}\left(1 + \frac{se}{k^2}B_{5\parallel}\right) \\
&\qquad \times \left[\theta(k_{\mathrm{F}} - k) + \frac{se}{2k}B_{5\parallel}\delta(k - k_{\mathrm{F}})\right]\hat{\boldsymbol{k}}
\end{aligned} \tag{E5}$$

$$= -s\frac{e^2 v_{\mathrm{F}}}{4\pi^2}k_{\mathrm{F}}\boldsymbol{B}_5 = -s\frac{e^2\mu}{4\pi^2}\boldsymbol{B}_5, \tag{E6}$$

which accounts for the chiral axial (pseudo) magnetic effect under the axial magnetic field $\boldsymbol{B}_5$, given by $\boldsymbol{J}_{\mathrm{eq}}^{(0)}$ in the main text.

$\boldsymbol{j}^{(0)\mathrm{a}}_{s\eta}$ can be obtained by evaluating the $\boldsymbol{k}$-space integral

$$\boldsymbol{j}^{(0)\mathrm{a}}_{s\eta} = -e\int \frac{d^3\boldsymbol{k}}{(2\pi)^3}\boldsymbol{R}^{(0)}_{s\eta}(\boldsymbol{k})\delta f^{(0)}_{s\eta}(\boldsymbol{k}) \tag{E7}$$

$$= -ev_{\mathrm{F}}\int \frac{d^3\boldsymbol{k}}{(2\pi)^3}\hat{\boldsymbol{k}}\left(1+\frac{se}{k^2}B_{5\parallel}\right)\delta f^{(0)}_{s\eta}(\boldsymbol{k}). \tag{E8}$$

In the integrand function

$$\left(1+\frac{se}{k^2}B_{5\parallel}\right)\delta f^{(0)}_{s\eta}(\boldsymbol{k}), \tag{E9}$$

the terms surviving after the angular integral can be extracted, by using the angular integral formulae Eq. (B9)-(B12), as

$$\frac{se^3\tau\tau_*}{k^3}\Big[-(\boldsymbol{E}_0\cdot\boldsymbol{B}_5)E_{0\parallel}\delta_{\mathrm{F}} + E_0^2B_{0\parallel}(-2\delta_{\mathrm{F}} - k\delta'_{\mathrm{F}}) + E_{0\parallel}^2B_{5\parallel}(5\delta_{\mathrm{F}} - 5k\delta'_{\mathrm{F}} + 2k^2\delta''_{\mathrm{F}})\Big]. \tag{E10}$$

By evaluating the angular and radial integrals, we obtain

$$\boldsymbol{j}^{(0)\mathrm{a}}_{s\eta} = \frac{se^4v_{\mathrm{F}}\tau\tau_*}{(2\pi)^3k_{\mathrm{F}}}\left[(\boldsymbol{E}_0\cdot\boldsymbol{B}_5)\frac{4\pi}{3}\boldsymbol{E}_0 + 2E_0^2\frac{4\pi}{3}\boldsymbol{B}_5 - 5\left(\frac{4\pi}{5}(\boldsymbol{E}_0\cdot\boldsymbol{B}_5)\boldsymbol{E}_0 - \frac{4\pi}{15}(\boldsymbol{B}_5\times\boldsymbol{E}_0)\times\boldsymbol{E}_0\right)\right] \tag{E11}$$

$$= -s\frac{e^4v_{\mathrm{F}}\tau\tau_*}{6\pi^2k_{\mathrm{F}}}(\boldsymbol{B}_5\times\boldsymbol{E}_0)\times\boldsymbol{E}_0, \tag{E12}$$

where we have used the vector triple product relation

$$(\boldsymbol{B}_5\times\boldsymbol{E}_0)\times\boldsymbol{E}_0 = (\boldsymbol{E}_0\cdot\boldsymbol{B}_5)\boldsymbol{E}_0 - E_0^2\boldsymbol{B}_5. \tag{E13}$$

$\boldsymbol{j}^{(0)\mathrm{b}}_{s\eta}$ is given by the $\boldsymbol{k}$-space integral

$$\boldsymbol{j}^{(0)\mathrm{b}}_{s\eta} = -e\int \frac{d^3\boldsymbol{k}}{(2\pi)^3}\sum_{\pm}\boldsymbol{R}^{(\pm\omega)}_{s\eta}(\boldsymbol{k})f^{(\mp\omega)}_{s\eta}(\boldsymbol{k}) \tag{E14}$$

$$= -s\eta e^2\boldsymbol{E}_0\times\int \frac{d^3\boldsymbol{k}}{(2\pi)^3}\,\hat{\boldsymbol{k}}\frac{1}{2k^2}\sum_{\pm}f^{(\mp\omega)}_{s\eta}(\boldsymbol{k}). \tag{E15}$$

In the integrand function

$$\frac{1}{2k^2}\sum_{\pm}f^{(\mp\omega)}_{s\eta}(\boldsymbol{k}), \tag{E16}$$

the term surviving after the angular integral can be extracted as

$$-\eta e^2v_{\mathrm{F}}\tau_{**}^2\frac{1}{k^3}\left(\boldsymbol{B}_5\times\boldsymbol{E}_0\right)_{\parallel}\delta_{\mathrm{F}}. \tag{E17}$$

By evaluating the angular and radial integrals, we obtain

$$\boldsymbol{j}^{(0)\mathrm{b}}_{s\eta} = \frac{se^4v_{\mathrm{F}}\tau_{**}^2}{(2\pi)^3k_{\mathrm{F}}}\boldsymbol{E}_0\times\frac{4\pi}{3}\left(\boldsymbol{B}_5\times\boldsymbol{E}_0\right) \tag{E18}$$

$$= -\frac{se^4v_{\mathrm{F}}\tau_{**}^2}{6\pi^2k_{\mathrm{F}}}\left(\boldsymbol{B}_5\times\boldsymbol{E}_0\right)\times\boldsymbol{E}_0. \tag{E19}$$

By collecting Eqs. (E12) and (E19), we obtain the final form given in the main text.
